# Proportion and clinical characteristics of non-asthmatic non-smokers among adults with airflow obstruction

**DOI:** 10.1371/journal.pone.0196132

**Published:** 2018-05-09

**Authors:** Hiroto Takiguchi, Tomoe Takeuchi, Kyoko Niimi, Hiromi Tomomatsu, Katsuyoshi Tomomatsu, Naoki Hayama, Tsuyoshi Oguma, Takuya Aoki, Tetsuya Urano, Satomi Asai, Hayato Miyachi, Koichiro Asano

**Affiliations:** 1 Division of Pulmonary Medicine, Department of Medicine, Tokai University School of Medicine, Isehara, Kanagawa, Japan; 2 Division of Medical Education, Department of Basic Medicine, Tokai University School of Medicine, Isehara, Kanagawa, Japan; 3 Department of Laboratory Medicine, Tokai University School of Medicine, Isehara, Kanagawa, Japan; National Yang-Ming University, TAIWAN

## Abstract

**Background and objectives:**

Chronic obstructive pulmonary disease (COPD) mainly develops after long-term exposure to cigarette or biomass fuel smoke, but also occurs in non-smokers with or without a history of asthma. We investigated the proportion and clinical characteristics of non-smokers among middle-aged to elderly subjects with airflow obstruction.

**Methods:**

We retrospectively analyzed 1,892 subjects aged 40–89 years who underwent routine preoperative spirometry at a tertiary university hospital in Japan. Airflow obstruction was defined as a forced expiratory volume in 1 second (FEV_1_)/forced vital capacity < 0.7 or as the lower limit of the normal.

**Results:**

Among 323 patients presenting with FEV_1_/forced vital capacity < 0.7, 43 had asthma and 280 did not. Among the non-asthmatic patients with airflow obstruction, 94 (34%) were non-smokers. A larger number of women than men with airflow obstruction had asthma (26% vs. 7.6%, p < 0.001), or were non-smokers among non-asthmatics (72% vs. 20%, p < 0.001). Non-asthmatic non-smokers, rather than non-asthmatic smokers, asthmatic non-smokers, and asthmatic smokers, exhibited better pulmonary function (median FEV_1_: 79% of predicted FEV_1_ vs. 73%, 69%, and 66%, respectively, p = 0.005) and less dyspnea on exertion (1% vs. 12%, 12%, and 28%, respectively, p = 0.001). Pulmonary emphysema on thoracic computed tomography was less common in non-smokers (p < 0.001). Using the lower limit of the normal to define airflow obstruction yielded similar results.

**Conclusions:**

There are a substantial number of non-smokers with airflow obstruction compatible with COPD in Japan. In this study, airflow obstruction in non-smokers was more common in women and likelier to result in mild functional and pathological abnormalities than in smokers. Further studies are warranted to investigate the long-term prognosis and appropriate management of this population in developed countries, especially in women.

## Introduction

Persistent airflow obstruction is the key feature of chronic obstructive pulmonary disease (COPD), one of the leading causes of morbidity and mortality worldwide [1]. This condition is associated with enhanced chronic inflammation, impaired repair mechanisms, and destruction of the airways and lungs in response to noxious gas and particles, especially those found in cigarette smoke, which is the leading risk factor for COPD [1, 2]. The total number of cigarettes smoked during one’s lifetime has a strong negative correlation with forced expiratory volume in 1 second (FEV_1_), regardless of other intrinsic and extrinsic factors [3]. Therefore, previous epidemiological and clinical studies of COPD or persistent airflow obstruction have focused on smokers [4–7].

Numerous studies, however, have shown that there are substantial numbers of non-smokers with obstructive pulmonary dysfunction worldwide. Among a sample of 12,980 lifelong never-smokers in the United States, 5.1% demonstrated persistent airflow obstruction [8]. Another study found that the proportion of never-smokers among patients with airflow obstruction ranged from 20% to 35% in the United States and Europe [6]. The proportion of non-smokers among patients with COPD is even higher in developing countries. Non-smokers account for 38% of patients with COPD in China, 48% in South Africa, and 69% in India [4, 6, 9]. A history of asthma, pulmonary tuberculosis, occupational exposure to gases and dusts, low socioeconomic status, and exposure to indoor or outdoor air pollution have been suggested as predisposing factors for COPD in non-smokers [5–7, 10, 11]. Exposure to household biomass smoke is particularly pertinent to the higher ratio of non-smokers with COPD among the residents of developing countries [12, 13].

There are significant differences in the clinical characteristics of COPD caused by cigarette smoke and the disease in non-smokers exposed to biomass fuel smoke. A small study comparing never-smokers and smokers with or without biomass smoke exposure suggested that COPD caused by biomass exposure is characterized by less pulmonary emphysema, more prominent air trapping, and a worse quality of life [14]. In contrast, little information is available about the clinical characteristics of COPD among non-smokers in developed countries, where people are unlikely to be exposed to biomass smoke or other forms of indoor air pollution. In this study, we retrospectively investigated the proportion and clinical characteristics of non-asthmatic non-smokers with airflow obstruction in a cohort of surgical patients who underwent routine preoperative spirometry at a tertiary university hospital in Japan.

## Materials and methods

### Study subjects and data collection

We retrospectively retrieved the medical records of adult patients aged ≥ 40 years who underwent spirometry between July 2009 and February 2010 for preoperative screening at Tokai University Hospital in Japan, where surgical patients were routinely evaluated through spirometry. The data collected included age; sex; body mass index; respiratory symptoms (cough and sputum production and dyspnea on exertion); history of asthma; cigarette smoking; and pharmacotherapy for respiratory conditions with inhaled bronchodilators, corticosteroids, theophyllines, or mucolytics [15]. Individuals aged ≥ 90 years were excluded from the analysis because their predicted values of pulmonary function were not available. “Non-smokers” were defined as never or former smokers with less than 5 pack-years. This protocol was approved by the Institutional Review Board of Tokai University Hospital (#14R-188). Consent was obtained by presenting an opt-out poster, and written consent was waived according to local ethics regulations and Ethical Guidelines for medical and health research involving human subjects in Japan. The research was conducted according to the principles of the Declaration of Helsinki.

### Spirometry and chest imaging

Spirometry was performed with a Super Spiro DISCOM-21FX III spirometer (CHEST Corp., Tokyo, Japan) by well-trained clinical technicians. Bronchodilators were not administered before measurement. The predicted values of vital capacity (VC), forced VC (FVC), and FEV_1_ for the Japanese population were calculated using an equation published in 2013 [16]. Airflow obstruction is defined as FEV_1_/FVC < 0.7, however, a fixed threshold of 0.7 for FEV_1_/FVC may lead to an underdiagnosis of COPD in the younger population and overdiagnosis in the elderly [17]. Therefore, we re-analyzed our data by defining airflow obstruction as FEV_1_/FVC < the lower limit of the normal (LLN), representing the age-specific fifth percentile of healthy non-smokers [16]. As a control, patients without airflow obstruction whose FEV_1_/FVC ranged from 0.70 to 0.74 and were higher than LLN were examined.

A history of asthma was confirmed by administering a questionnaire and conducting a medical interview. The history of other pulmonary diseases such as bronchiectasis or tuberculosis was ascertained by a review of medical charts.

Thoracic computed tomography (CT) images were analyzed by two experienced pulmonologists for the presence of pulmonary emphysema or fibrosis.

### Statistical analysis

All analyses were performed using EZR (Saitama Medical Center, Jichi Medical University, Saitama, Japan), which is a modified version of R Commander, a graphical user interface for R (The R Foundation for Statistical Computing, Vienna, Austria) designed to add statistical functions frequently used in biostatistics [18].

The patients were divided into four groups: 1) non-asthmatic non-smokers, 2) non-asthmatic smokers, 3) asthmatic non-smokers, and 4) asthmatic smokers. Numerical data are presented as medians and interquartile ranges, and categorical data as counts and percentages. Continuous variables were compared using the Kruskal–Wallis test, followed by multiple comparison analysis with the Steel test setting non-asthmatic non-smokers as the control. Trends in the prevalence of airflow obstruction according to age group were analyzed using the Cochran–Armitage test. For categorical variables, the chi-squared test or Fisher’s exact test was used, with Bonferroni’s correction for multiple comparisons. Logistic regression analysis was used to compare clinical characteristics of non-smokers and smokers, among either non-asthmatics or asthmatics, after adjustment for age, sex, and % predicted values of FEV_1_. p < 0.05 was considered statistically significant.

## Results

### Prevalence of airflow obstruction in adults aged 40–89 years

During the study period of 8 months, 1,892 adult patients aged 40–89 years (948 [50%] women, median age: 63 years) underwent preoperative spirometry, and 325 (17.0%) presented with airflow obstruction, defined as FEV_1_/FVC < 0.7. A significant association was observed between the prevalence of airflow obstruction and increased age: 11.3%, 26.2%, and 32.9% in men aged 40–59, 60–69, and 70–89 years (p < 0.001), respectively, and 5.2%, 12.9%, and 17.3% in women in the same age groups (p < 0.001).

When airflow obstruction was defined as FEV_1_/FVC < LLN, 295 patients (15.6%), including 28 with FEV_1_/FVC ≥ 0.7 (median age: 50 years), met this criterion, whereas 58 with FEV_1_/FVC < 0.7 (median age: 72 years) did not. The correlation between the prevalence of airflow obstruction and age was still observed in men (p < 0.001), but not in women (p = 0.3).

### Proportion of asthmatics among patients with airflow obstruction

**Fig 1:**
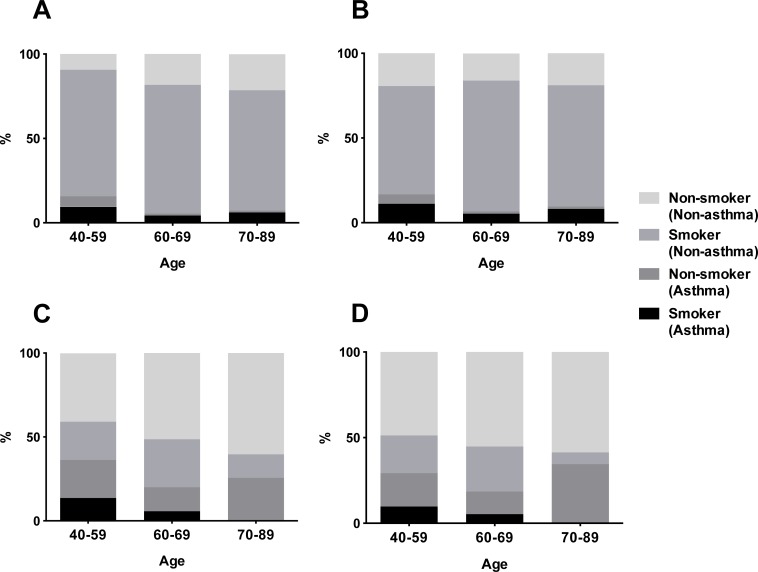
Proportions of non-smokers and smokers with or without self-reported asthma among patients with airflow obstruction. Proportions of non-asthmatic non-smokers, non-asthmatic smokers, asthmatic non-smokers, and asthmatic smokers among patients with airflow obstruction are shown according to age group in men (A and B) and women (C and D). Airflow obstruction was defined as FEV_1_/FVC < 0.70 (A and C) or FEV_1_/FVC < LLN (B and D).

Two patients were excluded from the analysis because of unavailability of detailed information about cigarette smoking, resulting in inclusion of 323 patients with FEV_1_/FVC < 0.7: 280 patients without asthma (14.8%) and 43 with asthma (2.3%). Non-smokers accounted for 34% of all patients without asthma, and 58% of all patients with asthma. The results were consistent when data from 48 patients with scheduled thoracic surgery for lung neoplasms or a history of lung tuberculosis or bronchiectasis, which might have affected their pulmonary functions, were removed from the analysis. Non-smokers accounted for 36% and 54% of non-asthmatic and asthmatic patients, respectively. Similarly, 293 patients with FEV_1_/FVC < LLN, namely 246 without asthma (13.0%) and 47 with asthma (2.5%), were analyzed. Non-smokers amounted for 37% and 57% of patients without and with asthma, respectively. Among all patients with airflow obstruction, the proportion of asthma was higher in women than in men (26% vs. 7.6%, p < 0.001), regardless of the age group (Fig 1).

In the subjects without airflow obstruction (FEV_1_/FVC ranging from 0.70 to 0.74), non-smokers accounted for 46% of patients without asthma (n = 216), which was significantly higher than in those with airflow obstruction (p < 0.01).

### Proportion of non-smokers among non-asthmatics with airflow obstruction

We focused on non-asthmatic patients with airflow obstruction. The proportion of non-smokers in this population varied according to age and sex. With respect to sex, non-asthmatic smokers accounted for 74% of male patients with airflow obstruction, across all age groups (Fig 1A). However, non-asthmatic non-smokers were prevalent (53%) in female, particularly in the older age groups (Fig 1C). When airflow obstruction was defined as FEV_1_/FVC < LLN, sex remained a factor significantly affecting the proportion of non-asthmatic non-smokers in patients with airflow obstruction (p < 0.001, Fig 1B and 1D).

### Clinical characteristics of non-asthmatic non-smokers with or without airflow obstruction

We first compared the clinical characteristics of non-asthmatic non-smokers with or without airflow obstruction. There was no difference in ages, but women predominated in the subjects without airflow obstruction. The proportion of patients who exhibited or had been treated with pharmacotherapy for respiratory symptoms was small in non-asthmatic non-smokers with airflow obstruction (Table 1), and these numbers were even smaller in those without airflow obstruction (S1 Table).

**Table 1:** Clinical characteristics according to smoking habits and history of asthma among patients with airflow obstruction, as defined by FEV_1_/FVC < 0.7.

	Non-asthma		Asthma		*p*
	Non-smoker	Smoker	Non-smoker	Smoker	
	(n = 94)	(n = 186)	(n = 25)	(n = 18)	
Age, years	70 (63, 76)	68 (62, 74)	69 (56, 75)	67 (55, 72)	0.18
Female	53 (56)	21 (11) *	21 (84)	5 (28)	< 0.001
Body-mass index, kg/m^2^	23 (21, 24)	22 (20, 24)	22 (20, 25)	21 (19, 24)	0.49
Smoking habit					
Pack-year	0 (0, 0)	40 (25, 50) *	0 (0, 0)	29 (17, 40) *	< 0.001
Non-smoker	94 (100)	0 (0) *	25 (100)	0 (0) *	< 0.001
Pulmonary function test					
FEV_1_/FVC, %	67 (64, 69)	65 (60, 68) *	65 (60, 67) *	63 (53, 65) *	< 0.001
FEV_1_, % predicted	79 (67, 87)	73 (63, 82) *	69 (43, 81) *	66 (59, 78) *	0.005
VC, % predicted	90 (79, 100)	87 (77, 97)	83 (69, 95)	84 (76, 101)	0.17
Thoracic CT scan	62 (66)	139 (75)	17 (68)	12 (67)	0.44
Pulmonary emphysema	4 (7)	83 (60) *	1 (6)	8 (67) *	< 0.001
Lung fibrosis	5 (8)	17 (12)	2 (12)	1 (8)	0.83
Respiratory disease manifestations					
Cough/sputum	7 (7)	23 (12)	4 (16)	4 (22)	0.25
Dyspnea	1 (1)	23 (12) *	3 (12)	5 (28) *	0.001
Pharmacotherapy					
On spirometry	5 (5)	22 (12)	15 (60) *	10 (56) *	< 0.001
After spirometry	9 (10)	62 (33) *	19 (76) *	17 (94) *	< 0.001

Values are medians (first quartile, third quartile) or numbers (%) of observations.

FEV_1_, forced expiratory volume in 1 second; FVC, forced vital capacity; VC, vital capacity; CT, computed tomography

p value was calculated for four groups.

* p < 0.05 compared with non-asthmatic non-smokers in multiple comparisons.

**Fig 2:**
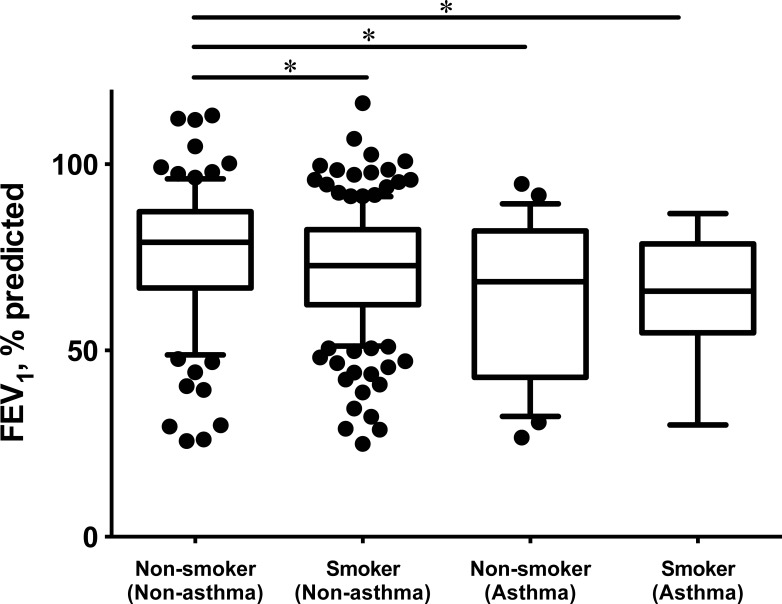
Box-and-whisker plot of the median FEV_1_ (% predicted) in patients with airflow obstruction. The median FEV_1_ (% predicted) was stratified by the presence or absence of a history of smoking and self-reported asthma. * p < 0.05.

We compared the clinical characteristics of non-asthmatic non-smokers with those of non-asthmatic smokers, asthmatic non-smokers, and asthmatic smokers (Table 1). No significant differences were observed in age or body mass index, but the number of women was higher in the non-asthmatic non-smoker group (56%) than in the non-asthmatic smoker group (11%). The median FEV_1_ (% predicted) was highest among non-asthmatic non-smokers (79%), followed by non-asthmatic smokers (73%), asthmatic non-smokers (69%), and asthmatic smokers (66%; p = 0.005; Fig 2). Respiratory symptoms, particularly dyspnea on exertion, were least common in non-asthmatic non-smokers (p = 0.001), although the proportion of patients undergoing pharmacotherapy was lowest in this population. Thoracic CT scans, which were performed for 230 of the patients with airflow obstruction (71%), demonstrated pulmonary emphysema less frequently in non-smokers, regardless of asthma (p < 0.001). Differences between non-asthmatic non-smokers and non-asthmatic smokers in the frequency of dyspnea on exertion or pulmonary emphysema on thoracic CT remained significant even after adjustment for age, sex, and FEV_1_. The findings were consistent when patients with scheduled thoracic surgery or a history of lung tuberculosis or bronchiectasis were excluded (S2 Table), or when airflow obstruction was defined as FEV_1_/FVC < LLN (S3 Table).

## Discussion

In a Japanese cohort of preoperative patients aged 40–89 years, 14.8% of patients exhibited airflow obstruction in the absence of self-reported asthma, and a third of whom were non-smokers. Compared with non-asthmatic smokers with airway obstruction, non-asthmatic non-smokers were (1) more likely to be women, (2) less likely to exhibit respiratory symptoms, and (3) less likely to exhibit pulmonary emphysema on thoracic CT. Non-smokers also tended to show milder airflow obstruction than smokers.

The proportion of non-smokers among patients with airflow obstruction varies among studies, ranging from 20% to 68%. In our data, 34% of non-asthmatic Japanese patients with FEV_1_/FVC < 0.7 were non-smokers. When an age-specific fifth percentile of FEV_1_ was used to define airflow obstruction, the proportion of non-smokers was even higher (37%). We found similar results in another cohort of 12,246 subjects aged ≥ 40 years who underwent spirometry at an annual health check-up. The prevalence of airflow obstruction was 13.8%, and the proportion of non-smokers was 36% among non-asthmatics with airflow obstruction (unpublished data).

According to our knowledge, this study is the first to analyze the proportion of non-smokers among Japanese subjects with airflow obstruction. However, the proportion can be estimated based on the results of two previous epidemiological studies in Japan, the Nippon COPD epidemiology study and Takahata study [19, 20]. The former study recruited 2,343 participants (age ≥ 40 years, mean = 58 years; 48% women and 47% non-smokers), and airflow obstruction was observed in 5.8% of never smokers and in 15.4% and 15.6% of former and current smokers, respectively, indicating that non-smokers accounted for 25% of all subjects with airflow obstruction [19]. A population-based study in Takahata city, which examined 2,917 participants (age ≥ 40 years, mean = 63 years; 55% women), found that 44% of subjects with airflow obstruction were never smokers [20]. These data are consistent with the proportion of non-smokers among subjects with airflow obstruction or COPD in other East Asian countries, such as Korea (33%) and China (38%), but relatively higher than the numbers obtained in the United States (25%) and Europe (20%–37%) [6].

A key characteristic among patients with airflow obstruction who had no history of asthma or cigarette smoking was female predominance. We observed that, after excluding patients with asthma, 72% of women with airflow obstruction were non-smokers, whereas only 20% of men in the same category were non-smokers. A similar trend was observed in the Takahata study, where 89% of women with airflow obstruction were non-smokers compared with only 25% of their male counterparts [20]. The sex difference is much smaller in Western countries; 26% of women and 19% of men with airflow obstruction were non-smokers in a cohort from Denmark [11]. Considering the indoor environment in Japan, this female predominance in non-smokers with airflow obstruction cannot be attributed to household biomass smoke exposure in women, as in developing countries [6]. The smoking rate among Japanese women is much lower than among men (percentage of former or current smokers: 10.5% for women and 79.5% for men) [21], and therefore, a higher rate of women exposed to environmental tobacco smoke is the probable explanation.

In contrast, milder disease manifestation in non-smokers with airflow obstruction was observed in both East Asian and Western countries. We found that non-smokers with airflow obstruction exhibited milder functional impairment of the lungs, fewer symptoms (particularly dyspnea on exertion), and less pulmonary emphysema visible on chest imaging, even with adjustment for pulmonary function. Our results are in concordance with the data from a population-based study from Denmark reporting that non-smokers with COPD demonstrate milder pulmonary function impairment, as well as fewer symptoms, such as cough and dyspnea on exertion, and have a better prognosis, than current and former smokers with COPD [11]. Another retrospective observational study from China, which examined patients hospitalized for the evaluation of lung tumors, reported similar results [22]. These studies, in addition to ours, suggest that not only impairment of pulmonary function but also long-term exposure to tobacco smoke are important factors in disease severity and prognosis of COPD.

The clinical characteristics of non-smokers with airflow obstruction in our study and in other studies conducted in developed countries are significantly different from those in developing countries, where people, particularly women, are frequently exposed to biomass fuel smoke. Absence or milder presentation of pulmonary emphysema is common between female never-smokers with COPD who had been exposed to biomass fuel smoke and non-smokers in our study [14]. However, the patients exposed to biomass smoke showed moderate to severe pulmonary function impairment equivalent to that in tobacco smokers and had even worse symptoms and quality of life scores [14]. Therefore, we may need to carefully discriminate between COPD in tobacco smokers, in non-smokers exposed to biomass fuel smoke, and in non-smokers without substantial exposure to indoor air pollution.

There were also substantial differences in the clinical characteristics of non-asthmatic non-smokers with airflow obstruction and patients with self-reported asthma who demonstrated airflow obstruction. Although female predominance was observed in both groups, asthmatics were accompanied by worse pulmonary dysfunction and more complaints of dyspnea, with a high rate of pharmacotherapy. These results are compatible with those in a previous report that asthmatic features contribute to impaired pulmonary function and quality of life [23, 24].

Our study has several limitations. First, this is a single-center, cross-sectional study. We are currently analyzing the rate of decline in pulmonary function in smokers and non-smokers using the annual health check-up database in our medical center, which includes data from more than 20,000 participants over a 10-year period. Second, there are selection biases because we recruited surgical patients who underwent preoperative spirometry, which could be one of the reasons why the prevalence of airflow obstruction was higher than that reported in population-based studies in Japan [19, 20]. Despite this limitation, the proportion and clinical characteristics of non-smokers among patients with airflow obstruction was equivalent between our study and previous studies, suggesting that “non-smoker COPD” has robust characteristics. Third, we analyzed only prebronchodilator FEV_1_, because post-bronchodilator FEV_1_ is not routinely measured in the evaluation of preoperative patients in clinical practice. Therefore, we could not label patients with airflow obstruction as definitive cases of COPD, although patients with asthma were carefully excluded and the analyses were performed based on two criteria for airflow obstruction. However, results similar to ours have been reported by other researchers who conducted post-bronchodilator spirometry [5, 22], suggesting that our results reflect the true proportion and clinical characteristics of non-smoker COPD. Fourth, a history of asthma was reported by some patients, but not diagnosed by physicians. Therefore, some patients may have been misclassified. Fifth, environmental tobacco smoke and other indoor/outdoor air pollution has been suggested as a possible cause of COPD, especially in non-smokers; however, we could not analyze any of these factors in this retrospective study.

## Conclusions

We confirmed that there is a substantial proportion of non-smokers with airflow obstruction compatible with COPD, even in the absence of concomitant asthma. In our study, non-asthmatic non-smokers with airflow obstruction were predominantly women and were likely to present with milder functional and pathological abnormalities than were COPD patients with a history of smoking. These subjects were mostly untreated because non-smokers have been excluded from clinical trials of anti-COPD drugs and little is known about the clinical benefits of therapeutic intervention. The proportion and clinical characteristics of non-smokers with airflow obstruction seem to be relatively consistent among developed countries except for sex ratio, but substantially different from those in developing countries. Further studies are warranted to investigate the long-term prognosis and appropriate management of COPD in non-smokers.

## Supporting information

S1 Table Clinical characteristics of non-asthmatic non-smokers with or without airflow obstruction. (DOCX)

S2 Table Clinical characteristics of patients with airflow obstruction (FEV_1_/FVC < 0.7) in the absence of scheduled thoracic surgery or a history of lung tuberculosis or bronchiectasis. (DOCX)

S3 Table Clinical characteristics according to smoking habits and history of asthma among patients with airflow obstruction, as defined by the FEV_1_/FVC < LLN. (DOCX)
