# Supplementary material for: Proportion and clinical characteristics of non-asthmatic non-smokers among adults with airflow obstruction
Source: PLoS One. 2018 May 9;13(5):e0196132. doi: 10.1371/journal.pone.0196132 (PMC5942827; doi:10.1371/journal.pone.0196132)
Supplement: S1 Table — (DOCX) [file pone.0196132.s001.docx]

**S1 Table. Clinical characteristics of non-asthmatic non-smokers with or without airflow obstruction**

Values are medians (first quartile, third quartile) or numbers (%) of observations.

FEV1, forced expiratory volume in 1 second; FVC, forced vital capacity; VC, vital capacity;

CT, computed tomography; LLN, lower limit of the normal

|  | Non-asthmatic non-smokers | | *p* |
| --- | --- | --- | --- |
|  | 0.7 ≤ FEV_1_/FVC < 0.74  and FEV_1_/FVC > LLN | FEV_1_/FVC < 0.7 |  |
|  | n = 99 | n = 94 |  |
| Age, years | 69 (61, 75) | 70 (63, 76) | 0.22 |
| Female | 74 (75) | 53 (56) | 0.007 |
| Body-mass index, kg/m^2^ | 22 (21, 24) | 23 (21, 24) | 0.51 |
| Pulmonary function test |  |  |  |
| FEV_1_/FVC, % | 73 (72, 73) | 67 (64, 69) | < 0.001 |
| FEV_1_, % predicted | 89 (82, 97) | 79 (67, 87) | < 0.001 |
| VC, % predicted | 95 (87, 102) | 90 (79, 100) | 0.02 |
| Thoracic CT scan | 44 (45) | 62 (66) | 0.004 |
| Pulmonary emphysema | 0 (0) | 4 (7) | 0.13 |
| Lung fibrosis | 2 (4) | 5 (8) | 0.70 |
| Respiratory disease manifestations | |  |  |
| Cough/sputum | 1 (1) | 7 (7) | 0.03 |
| Dyspnea | 4 (4) | 1 (1) | 0.37 |
| Pharmacotherapy |  |  |  |
| On spirometry | 0 (0) | 5 (5) | 0.03 |
| After spirometry | 0 (0) | 9 (10) | 0.001 |
