# Supplementary material for: Proportion and clinical characteristics of non-asthmatic non-smokers among adults with airflow obstruction
Source: PLoS One. 2018 May 9;13(5):e0196132. doi: 10.1371/journal.pone.0196132 (PMC5942827; doi:10.1371/journal.pone.0196132)
Supplement: S2 Table — (DOCX) [file pone.0196132.s002.docx]

**S2 Table. Clinical characteristics of patients with airflow obstruction (FEV_1_/FVC < 0.7) in the absence of scheduled thoracic surgery or a history of lung tuberculosis or bronchiectasis**

|  | Non-asthma | |  | Asthma | | *p* |
| --- | --- | --- | --- | --- | --- | --- |
|  | Non-smoker | Smoker |  | Non-smoker | Smoker |  |
|  | n=86 | n=152 |  | n=20 | n=17 |  |
| Age, years | 69 (63, 76) | 67 (62, 74) |  | 66 (55, 72) | 66 (54, 72) | 0.086 |
| Female | 48 (56) | 14 (9) * |  | 17 (85) | 5 (29) | < 0.001 |
| Body-mass index, kg/m^2^ | 23 (21, 24) | 22 (20, 24) |  | 22 (20, 27) | 21 (19, 25) | 0.59 |
| Smoking habit |  |  |  |  |  |  |
| Pack-year | 0 (0, 0) | 40 (24, 50) * |  | 0 (0, 0) | 30 (16, 40) * | < 0.001 |
| Non-smoker | 86 (100) | 0 (0) * |  | 20 (100) | 0 (0) * | < 0.001 |
| Pulmonary function test |  |  |  |  |  |  |
| FEV_1_/FVC, % | 67 (65, 69) | 66 (60, 68) * |  | 66 (65, 68) | 63 (54, 66) * | 0.002 |
| FEV_1_, % predicted | 80 (69, 87) | 75 (65, 85) |  | 72 (59, 83) | 66 (64, 78) | 0.046 |
| VC, % predicted | 91 (80, 101) | 88 (79, 98) |  | 84 (74, 98) | 84 (79, 102) | 0.48 |
| Thoracic CT scan | 54 (63) | 107 (70) |  | 13 (65) | 11 (65) | 0.67 |
| Pulmonary emphysema | 4 (7) | 64 (60) * |  | 0 (0) | 7 (64) * | < 0.001 |
| Lung fibrosis | 4 (7) | 14 (13) |  | 0 (0) | 1 (9) | 0.41 |
| Respiratory disease manifestations | | |  |  |  |  |
| Cough/sputum | 4 (5) | 20 (13) |  | 3 (15) | 3 (18) | 0.15 |
| Dyspnea | 1 (1) | 19 (13) * |  | 1 (5) | 5 (29) * | 0.001 |
| Pharmacotherapy | 3 (4) | 18 (12) |  | 10 (50) * | 9 (53) * | < 0.001 |

Values are medians (first quartile, third quartile) or numbers (%) of observations.

FEV_1_, forced expiratory volume in 1 second; FVC, forced vital capacity; VC, vital capacity; CT, computed tomography

*p* value was calculated for four groups

* p < 0.05 compared with non-asthmatic non-smokers in multiple comparisons.
