# Supplementary material for: Proportion and clinical characteristics of non-asthmatic non-smokers among adults with airflow obstruction
Source: PLoS One. 2018 May 9;13(5):e0196132. doi: 10.1371/journal.pone.0196132 (PMC5942827; doi:10.1371/journal.pone.0196132)
Supplement: S3 Table — (DOCX) [file pone.0196132.s003.docx]

**S3 Table. Clinical characteristics according to smoking habits and history of asthma among patients with airflow obstruction, as defined by FEV1/FVC < LLN**

|  | Non-asthma | |  | Asthma | | *p* |
| --- | --- | --- | --- | --- | --- | --- |
|  | Non-smoker  (n = 91) | Smoker  (n = 155) |  | Non-smoker (n = 27) | Smoker  (n = 20) |  |
|  |  |  |  |  |  |  |
| Age, years | 64 (59, 72) | 67 (61, 73) |  | 66 (54, 72) | 66 (48, 72) | 0.41 |
| Female | 58 ( 64) | 21 (14) * |  | 23 (85) | 6 (30) | < 0.001 |
| Body mass index, kg/m^2^ | 22 (20, 24) | 22 (20, 24) |  | 22 (20, 25) | 21 (19, 25) | 0.75 |
| Smoking habit |  |  |  |  |  |  |
| Pack-years | 0 (0, 0) | 40 (25, 51) * |  | 0 (0, 0) | 25 (16, 40) * | < 0.001 |
| Non-smoker | 91 (100) | 0 (0) * |  | 27 (100) | 0 (0) * | < 0.001 |
| Pulmonary function test |  |  |  |  |  |  |
| FEV_1_/FVC, % | 66 (63, 69) | 64 (59, 67) * |  | 65 (61, 68) | 64 (54, 66) * | 0.001 |
| FEV_1_, % predicted | 78 (66, 86) | 72 (61, 82) |  | 70 (43, 80) * | 69 (62, 81) | 0.021 |
| VC, % predicted | 89 (78, 101) | 87 (78, 97) |  | 81 (71, 95) | 88 (78, 102) | 0.079 |
| Thoracic CT scan | 53 (58) | 111 (72) |  | 17 (63) | 12 (60) | 0.17 |
| Emphysema | 6 (11) | 67 (60) * |  | 1 (6) | 8 (67) * | < 0.001 |
| Fibrosis | 3 (6) | 9 (8) |  | 2 (11) | 1 (8) | 0.88 |
| Respiratory disease manifestations | |  |  |  |  |  |
| Cough/sputum | 5 (6) | 20 (13) |  | 4 (15) | 5 (25) | 0.062 |
| Dyspnea | 1 (1) | 22 (14) * |  | 4 (15) | 5 (25) * | 0.002 |
| Pharmacotherapy | 4 (4) | 19 (12) |  | 14 (52) * | 10 (50) * | < 0.001 |

Values are medians (first quartile, third quartile) or numbers (%) of observations.

FEV_1_, forced expiratory volume in 1 second; FVC, forced vital capacity; VC, vital capacity; CT, computed tomography.

p value was calculated for four groups.

* p < 0.05 compared with non-asthmatic non-smokers in multiple comparisons.
